# The Effects of Environmental Enrichment on the Physiology, Behaviour, Productivity and Meat Quality of Pigs Raised in a Hot Climate

**DOI:** 10.3390/ani9050235

**Published:** 2019-05-13

**Authors:** Emma Fàbrega, Míriam Marcet-Rius, Roger Vidal, Damián Escribano, José Joaquín Cerón, Xavier Manteca, Antonio Velarde

**Affiliations:** 1 Animal Welfare Program, IRTA, Veïnat de Sies s/n, 17121 Monells, Spain; cvr.milan42@gmail.com (R.V.); antonio.velarde@irta.cat (A.V.); 2 Physiological and Behavioural Mechanisms of Adaptation Department, IRSEA (Research Institute in Semiochemistry and Applied Ethology), Quartier Salignan, 84400 Apt, France; m.marcet@group-irsea.com; 3 Interdisciplinary Laboratory of Clinical Analysis (Interlab-UMU), Regional Campus of International Excellence ‘Campus Mare Nostrum’, University of Murcia, Campus de Espinardo s/n, Espinardo, 30100 Murcia, Spain; det20165@um.es (D.E.); jjceron@um.es (J.J.C.); 4 Department of Animal and Food Science, School of Veterinary Science, Autonomous University of Barcelona, 08193 Bellaterra (Barcelona), Spain; xavier.manteca@uab.cat

**Keywords:** environmental enrichment, pig, behaviour, performance, Chromogranin-A, lactate, skin lesions, meat quality

## Abstract

**Simple Summary:**

European Union (EU) legislation states that the routine tail docking of pigs should not be carried out and that manipulable materials should be made available to all pigs to prevent tail biting and allow them to behave naturally. However, between 90 and 95% of pigs within the EU still have their tails docked to avoid the risk of tail biting. Farmers say they require information tailored to their particular production systems before they abandon this practice. In this study, four types of enrichment materials used in Spanish pig fattening production systems are compared. Most of these systems have fully slatted floors and high external temperatures for considerable periods of the year. The effects of chains (the control group), wood, paper or straw in a rack on the behaviour, health/physiology, performance and meat and carcass quality are evaluated. Straw in a rack was found to be the best material to meet the behavioural needs of pigs, whereas paper met the criteria of being manipulable, but only for a short period. To avoid the risk of blockages in the slurry system, there are some practical issues to consider and improvements to be made to the design of the rack for providing straw used in this study.

**Abstract:**

Some positive effects regarding the use of enrichment material on the stimulation of pig exploration and a reduction in redirected behaviour was reported. This study aims to evaluate the effects of four enrichment materials on the behaviour, physiology/health, performance and carcass and meat quality in pigs kept in Spanish production conditions. Ninety-six male pigs (six pigs/pen) ranging from 70 to 170 days old were used. Chains were used for the control group (CH), and wooden logs (W), straw in a rack (S) or paper (P) were also used. The pigs were subjected to two pre-slaughter treatments: 0 or 12 h of fasting. Their behaviour was observed for 12 weeks using scan and focal sampling. Samples of the Neutrophil: Lymphocyte (N:L) ratio and lactate were obtained from the pigs at 66 and 170 days old. Saliva samples for Chromogranin-A (CgA) were obtained at 67, 128, 164 and 170 days old. The weight, skin lesions and feed intake of the pigs were recorded. S triggered more exploratory behaviour than W and CH (*P* < 0.001). Skin lesions and redirected behaviour were lower for pigs with S (*P* < 0.01 and P < 0.05, respectively). The pigs offered S presented lower CgA after no fasting than pigs with P or CH (*P* = 0.055). Lactate was higher in pigs with W and CH treatments, regardless of fasting (*P* < 0.05). The N:L ratio increased over time (*P* < 0.05). No other significant effects were found. Overall, straw in a rack was the enrichment material that enhanced pig inherent behaviour.

## 1. Introduction

Environmental enrichment has been defined as the improvement in the biological functioning of captive animals resulting from modifications to their environment [1]. Enriched environments aim to improve the welfare of animals by allowing them to perform more of their species-specific behaviour. Pigs kept in intensive production systems are exposed to several management or environmental conditions which do not always meet their individual requirements. Specifically, pigs in many intensive husbandry systems are often not provided with the proper foraging materials which can lead to stress, because they normally spend most of their active time rooting if given the possibility [2]. The lack of proper enrichment materials to allow them to express their foraging behaviour may also lead to frustration (for a review see [3,4,5]) and has been found to be an underlying cause of tail biting [6,7,8,9]. The practice of tail docking has been adopted for a long time and is common in pig farms to prevent the risk of tail biting, although it does not always work and outbreaks may still occur in docked pigs [4,8,9].

The current European Union (EU) Directive for the protection of pigs [10] stipulates that enrichment materials, such as straw or other suitable materials, should be provided to satisfy the behavioural needs of all categories of pigs (i.e., to satisfy both rooting and nesting behavioural needs). Furthermore, the directive [10] specifies that tail docking should not be routinely carried out and stipulates the measures that farmers should take before they resort to docking. Although the legal and social demands to avoid tail docking are increasing, recent studies show that 90–95% of pigs in the EU still have their tails docked to avoid the risk and consequences of tail biting [11,12]. Farmers say they are reluctant to stop tail docking for the following reasons: (1) the solutions are not straightforward because the reasons why an outbreak of tail biting develops, even in docked pigs, are complex (2) the fear and frustration of failure if they move to raising pigs with intact tails; (3) the economic consequences of both the outbreaks and of implementing prevention measures that may not work. Therefore, the EU is investing its efforts in disseminating the existing information on how to prevent tail biting and conducting research into new strategies, such as innovations in environmental enrichment. One important consideration is how the existing knowledge can be applied in various farming and climatic conditions in different countries. One of the objectives of this study is to evaluate the effects of different enrichment materials on docked pigs in the Spanish climate, as a first step towards increasing farmers’ confidence in strategies that could allow them to move towards raising pigs with intact tails.

It has been suggested that the availability of enrichment materials, especially straw, may increase the general activity, reduce stereotypes and increase the social interactions of the pigs [3,13]. Moreover, other positive effects have been reported, such as a reduced incidence of belly nosing, tail and ear biting, improved learning capabilities and reduced fearfulness [13,14,15,16]. Van de Weerd and Day [3] suggest that to be successful the enrichment material should meet four criteria: (1) it should increase species-specific behaviour, (2) it should maintain or improve levels of health, (3) it should improve the economics of the production system, and (4) it should be practical to employ. Scientific literature suggests that straw and similar substrate materials provided on the floor have the greatest potential to meet the first criteria, which addresses pig welfare [3,17]. However, in Spain most fattening pigs are kept on fully or partially slatted floors, and the liquid manure systems limit the use of substrate enrichments due to the possibility of blockages in the slurry system (i.e., the practicality criteria would not be met). Moreover, the high temperature conditions may prevent the use of large amounts of straw during certain periods of the year as this could cause heat stress. Therefore, point source object enrichment (i.e., ’objects limited in size and restricted to certain pen location(s)‘, as defined in [3]) may be more appropriate for slatted or partially slatted flooring production systems. Some studies have presented the potential beneficial effects of using point source materials [13,18,19], but their effectiveness varies depending on the type and properties of the material provided, as well as its position, the sense of novelty, cleanliness, interaction with management or climatic conditions and other attributes [3]. Some studies in fattening pigs have demonstrated that the use of ample, fresh wood attached horizontally to chains, as used in this study, can increase object manipulation and reduce both tail and ear damage when compared with single chains, branched chains or plastic objects [18]. Research into the effects of enrichment materials has often focused on behavioural outcomes, but it is also important to evaluate other aspects such as their effect on physiology, health, productivity, economic viability and practicability for farmers, and even the potential influence on the product quality [3,20]. Most objects have been considered insufficient on their own in having a positive effect on all the parameters. The EU Recommendation 2016 [21] classifies enrichment material as “optimal”, “suboptimal” or “marginal”, according to the level of interest, and provides guidance on how to combine them to optimise their efficacy. However, the list is not exhaustive and many aspects of how to enrich pens properly, especially fully slatted ones, are yet to be revealed.

The aim of this study is to evaluate the effect of four different types of enrichment material, feasible to implement in Spanish pig housing and climate conditions on: (1) behaviour; (2) physiology and health indicators; (3) productivity and (4) carcass and meat quality, in fattening docked pigs. The four criteria suggested by Van der Weerd and Day [3] have been used to evaluate the possibility of success of the four different types of enrichment material in the Spanish pig husbandry scenario.

## 2. Materials and Methods

### 2.1. Ethical Considerations

The housing, husbandry and use of animals for the procedures described in this paper was carried out in accordance with Spanish (RD 53/2013) and European legislation regarding animal experimentation and the care of animals under study. The project, including the experimental procedure, was approved by the Institute of Agri-food Research and Technology (IRTA) Ethics Committee and received a grant and approval (nº AGL2015-68373-C2-2-R) from the Spanish Ministry of Economy and Enterprises (MINECO). The authors acknowledge that the Commission staff working document accompanying the EU Recommendation 2016 [21] classifies the materials used in this experiment as suboptimal or marginal. This means that according to the commission working document, chains are considered as marginal enrichment materials, and therefore to fulfil the behavioural needs of the pigs on the farm must be complemented with either optimal or suboptimal materials. In this experiment, permission from the ethical committee was obtained to use chains in the ‘control’ group. Other materials used in this experiment come under the category of suboptimal to marginal, as ‘wood’ is considered suboptimal or marginal depending on its softness, as are paper and straw in a rack. According to the EU Recommendation 2016 [21], all these materials should be combined with other materials to complement their properties. However, the aim of this study is to evaluate the effects of these materials when isolated, and to avoid possible confounding results from their combination. A preliminary trial was conducted as part of this research which indicated that using some of the enrichment items individually under similar conditions, led to a considerable occurrence of tail biting. Therefore, due to ethical concerns, the authors decided to compare the efficacy of those materials (wood, chains) with new ones (straw in a rack) in docked pigs, as the first step towards moving to undocked pigs.

### 2.2. Animals and Housing and Experimental Conditions

Ninety-six male pigs ((Landrace, Large white) and Pietrain) were used in this experiment. The piglets were all born in a commercial pig farm within five days, from which two piglets/litter of similar weight were chosen. All pigs had their tails docked before they were seven days old. The pigs selected for this study arrived at the experimental farm with an average live weight of 23.50 ± 2.5 kg (mean ± SD) and were 60 days old. They were kept together before the start of the experiment until they were 70 days old and had an average weight of 26.1 ± 3.00 kg. The pigs were randomly divided into four treatments (24 pigs/treatment), as explained in Section 2.3. They were housed in groups of six in two identical rooms, with eight pens in each room (i.e., two pens of each enrichment type in each room). For differentiation purposes, each pig was allocated a different colour tag (blue, yellow, red, orange, green and white). The pigs were also identified by means of an electronic ear transponder. The pens were identical except for the enrichment material provided. The stocking density was 0.9 m^2^/pig, and the floors were fully slatted (commercial size: 80 mm wide slats with an 18 mm gap between them). There was one bowl-type drinker and one hopper available. The pens were divided by metallic fences which allowed the pigs to see each other and there was a solid metallic barrier in the lower part of the fence to prevent the enrichment materials moving between pens. The room was controlled by an automatic control system that regulated both temperature and ventilation. The room temperature was maintained at 22 ± 5 °C. The pigs were fed on a commercial pig diet provided ad libitum and they had continuous access to drinking water. This diet consisted of a commercial concentrate provided in a phased feeding regime (15.04% crude protein and 2.321 Kcal net energy at mid fattening). The experiment lasted for approximately three months, starting at the end of February and ending in mid-June, when the outside maximum temperature had already reached 30 °C.

### 2.3. Experimental Treatments

The four enrichment materials provided were chosen because they were feasible for use with fully slatted floors and in hot climates, and on the assumption that their practicality would encourage farmers to use them and combined when necessary. They consisted of (a) chains (considered as the control group); (b) wooden logs attached to a chain; (c) straw; and, (d) paper. For the chain treatment, two chains (50 cm long) were attached perpendicularly to the metal fence bars of each pen. One was attached to the door and another was attached to the same fence at a distance of approximately 50 cm. For the wood treatment, two wooden logs (30 × 5 × 5 cm) were hung perpendicularly from a chain, in the same positions as described for the chain treatment. Both the chains and wooden logs were near the floor but did not touch it. The wooden logs were made from fresh wood from the ash tree and were replaced after 20 days of use. Non-chopped straw was continuously available in a rack (60 × 40 × 80 cm), without a tray underneath to collect any spare straw. New straw was added once a day in the morning to ensure that the racks were completely full. For the paper treatment, two entire newspaper sheets (40 × 30 cm) were provided for each pig every day, one in the morning at around 8:30h and the other at midday around 14:00h.

### 2.4. Behavioural Observations

Instantaneous scan sampling and continuous focal sampling as described by Martin and Bateson [22] was used to record behaviour using an ethogram (as illustrated in Table 1). Once a week, for 12 weeks, two trained observers carried out direct observations of all the pens in each room in sessions of approximately four consecutive hours (from 10:00h. to 14:00h). Behavioural observations started when the pigs were 77 days old (one week after the provision of the enrichment treatments) and ended at 164 days (i.e., after three months of observations). Before starting the experimental procedure, the observers carried out preliminary trials to test their repeatability (a coefficient of correlation r > 0.8, *P* < 0.001 at the start of the procedure). The two observers carried out their observations simultaneously, one in each room, to avoid possible differences of time between rooms. Each group of four pens was scanned at seven-minute intervals, and focal samplings were recorded at three minutes per pen between two consecutive scan samplings (i.e., instantaneous scan samplings of activity were performed for two minutes at the beginning and end of each observation round and three minutes of focal sampling was performed in between, with two minutes rest). The same procedure was carried out for the other group of four pens in the same room. This scan and focal sampling interval was adapted from a similar methodology used in a previous study to suit the conditions of this experiment [13]. Thus, each observation day provided a total of 12 scans per animal and nine minutes of focal sampling per pen, divided into three periods of three minutes. Every week, the observers swapped rooms with each other and changed the order of observation of the eight pens within each room so they each observed all the rooms at different times. The behaviour observed according to each observational methodology is summarised in Table 1. Ten minutes before starting the observations, each observer entered the room and walked around for five minutes to allow the pigs to get used to their presence. Then, the observer moved towards the centre of the room, and remained there for another five minutes before starting the observation. The observation was carried out from the centre of the room from where all the pens could be seen.

### 2.5. Physiological Parameters

Saliva samples were obtained to analyse salivary Chromogranin-A (CgA), which has previously been reported as an indicator of acute and/or chronic stress [23,24]. The first saliva sample was taken when the pigs were 67 days old, three days before the enrichment materials were provided, to measure the basal levels. Two other samples were obtained at 128 and 164 days of age (approximately mid-procedure and end, and 1.5 and three months after providing the enrichment materials). A fourth saliva sample was collected at the slaughterhouse (170 days old), during lairage. Saliva samples were taken between 08:00 h and 11:00 h., by allowing the animals to chew on a cotton bud (Salivette^®^; SARSTEDT AG & Co., Nürbrecht, Germany) for more than 30 seconds and randomising the animals from different treatments. Cotton buds were offered using a clamp, with no restraint of the pigs. The sampling time was fixed up to a maximum of five minutes. Once sampled, and before the analysis, the salivettes^®^ were centrifuged at 3500 rpm for 10 min, the cotton buds were removed, and the tubes were frozen at −20 °C. Salivary CgA was measured using a validated time-resolved immunofluorometric assay (TR-IFMA) [23,24].

Blood samples were collected to perform a hemogram and determine the ratio neutrophil: lymphocytes (N:L) as an indicator of the immune system response and potential indicator of stress. Lactate was also measured in the second blood sample. The basal sample of blood was collected at 66 days of age (four days before providing enrichment) via a jugular vein puncture between 08:00 and 11:00 a.m. The pigs were gently restrained by one trained operator to obtain the samples, and the procedure took less than five minutes for all pigs. Samples were obtained at 170 days of age just after exsanguination at the slaughterhouse. The blood samples were collected in ethylene diamine tetra acetic acid (EDTA) (1 mg/mL) coated test tubes. Once sampled, the blood was refrigerated and immediately sent to the laboratory for basic haematology analysis. Another blood sample was collected at the slaughterhouse in EDTA tubes and immediately used to measure lactate using the commercial kit Lactate Scout-4^®^ (Biolaster, Gipuzkoa, Spain).

### 2.6. Performance Indicators and Body Lesions

The weight of the pigs was recorded when they were 68 days old (before starting the experiment), and then at 90, 107, 129, 157 and 168 days using a cage with a scale (MBWA100 Meier-Brakenberg; GmbH & Co, Extertal, Germany). Feed intake was recorded at pen level using a trolley equipped with scales (VLIEBO, Veenendal, The Netherlands), which allowed the amount of food provided to each pen to be recorded. When the pigs were weighed individually, the food left in the hopper was also weighed to allow for estimates of food intake by periods. The feed conversion ratio was estimated from the mean average daily weight gain and mean daily feed intake per pen (i.e., total pen feed intake/6 pigs per pen).

Skin lesions were assessed the day before the pigs were weighed in their home pens. The total number of lesions on one side of the pig in each one of five regions (ears, front, middle, rear -including tail- and legs) as defined in the Welfare Quality^®^ [25] was recorded.

### 2.7. Fasting Treatments and Carcass and Meat Quality Parameters

All the animals were slaughtered in a commercial abattoir at the age of 24 weeks (170 days). The mean live weight was 114.92 ± 11.02 kg. All pigs were gas stunned before sticking. The pigs were subjected to two different periods of on farm fasting, to provide different pre-slaughter stress conditions. The pigs in half of the pens per treatment, equally distributed in the two rooms, were fasted for 12 h and half were not fasted (i.e., two pens/enrichment and fasting treatment, 12 pigs/enrichment and fasting treatment). Transport to the slaughterhouse took 30 min and the pigs were kept in lairage for two hours before slaughter. They were gently handled when being loaded and unloaded to avoid mixing with unfamiliar pigs in the truck. The carcass weight was measured at 45 min *post mortem*, and the carcass yield was calculated by dividing the carcass weight by the live body weight. Furthermore, the backfat (LR3/4FOM) and muscle thicknesses (MFOM) were measured between the third and fourth last ribs, at 6 cm from the midline, using a Fat-O-Meat’er probe (Frontmatec A/S, Odense, Denmark). The official Spanish equation was used to calculate the lean percentage from the backfat and muscle thickness measurements. A ruler was used 24 h *post mortem* to measure the left carcass of each animal: (a) minimum fat and (b) skin thickness (perpendicular to the skin) over the *gluteus medius* muscle (MLOIN). A tape was used to measure the loin (from the atlas to the first lumbar vertebra) and carcass length (the first rib to the anterior edge of the pubic symphysis).

A Crison portable meter (Crison, Barcelona, Spain) equipped with a Xerolyte electrode and a Pork Quality Meater (PQM-I, INTEK Aichach, Germany) were used 24 h *post mortem* to measure muscle pH and electrical conductivity in the muscle *Longissimus thoracis* (LT) at the last rib level and Semimembranosus (SM), respectively. Luminosity L*, tendency to red a* and tendency to yellow b* (colour parameters on the CIELab space) were obtained using a Minolta Chromometer (CR-400, Minolta Inc., Osaka, Japan).

### 2.8. Statistical Analysis

All the statistical analyses were conducted using the Statistical Analysis System (SAS version 9.2; SAS institute Inc., Cary, NC; USA). Significance was established at *P* < 0.05, and the exact P value is provided for all those results with a *P* < 0.1. The results are presented as mean ± standard deviation (SD) or SE (standard error) unless otherwise indicated. The Shapiro-wilk test (with PROC UNIVARIATE in SAS) was used to examine the normality of the distributions. The experimental unit for the lesions, physiological indicators and carcass and meat quality parameters was individual, whereas for the behavioural observations and productivity data (weight, feed intake and food conversion ratio) the pen was considered as the experimental unit. The least square means of fixed effects (LS-means) with Tukey adjustment was used for comparisons when analysis of the variance indicated differences (*P* < 0.05).

The productivity parameters and physiological indicators presented a parametric distribution and were analysed using a generalised linear mixed model for repeated measurements (MIXED procedure and the covariance matrix Autoregressive AR(1) in SAS). The AR(1) covariance matrix was chosen because according to Schwarz’s Bayesian criterion it was the best fit for the model. The enrichment treatment and time and the interaction were considered as fixed effects, while the pen was considered as a random effect for the physiological indicators. For the weight analysis, the weight before starting the experiment was considered a covariate. The carcass and meat quality parameters were presented as a parametric distribution and were analysed using a general linear model (GLM), using enrichment treatment, fasting time and their interaction as fixed factors. Carcass weight was considered a covariate in those parameters where it was proved to have a significant effect. The lactate and CgA data collected at the slaughterhouse after two on farm fasting periods was also analysed using a GLM model with enrichment type, fasting time and its interaction as fixed effects.

The skin lesions and behavioural data were analysed using non-parametric generalised linear models for repeated measurements (GLIMMIX or GENMOD procedure, respectively). The Poisson distribution was used and for some parameters a negative binomial, depending on the deviance [26]. Lesions before providing enrichment were considered as a covariate. Enrichment material and time, and its interaction were used as fixed effects, and the pen as a random effect for skin lesions. The behavioural data from the scan samples was analysed as a percentage of the scans in each category in relation to the total number of scans per day, while data from focal sampling was analysed as the total number of counts observed in each category per pen and day. In the first analysis all 12 observations over time were included as a time effect on the behavioural data using a non-parametric generalised linear model (GENMOD procedure) with repeated measurements and the covariance matrix AR(1). An effect of time was observed, but the effect was the same as when averaging the data in three periods (i.e., four observations per period). Therefore, for simplicity, the results are presented for the averaged results in three periods, and not for the 12 observations over time.

## 3. Results

### 3.1. Behavioural Observations

**Figure 1:**
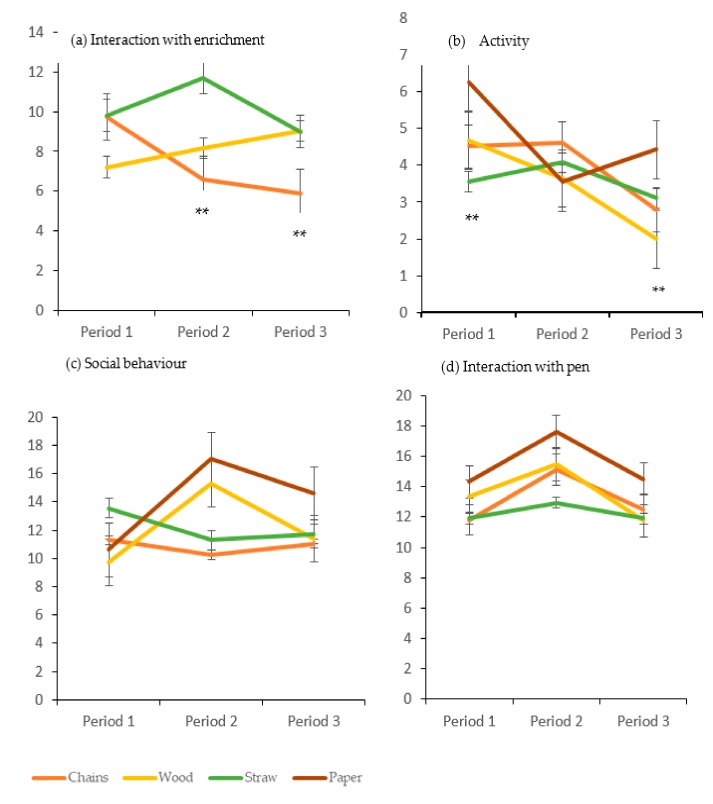
Behaviours observed by scan sampling (Mean percentage and SE) for the 4 types of enrichment materials. 1-3: averaged observations for first, second and third month of observations. ** *P* < 0.01 (see text for explanation of statistical differences).

Considering the four treatments, time had a significant effect on the lying behaviour, being a percentage higher in the third period compared to the other two periods in all four treatments (average % for all treatments: Period 1 = 46.90 ± 12.25; Period 2 = 47.51 ± 15.25 and Period 3 = 52.34 ± 15.70, χ^2^ (Chi-Square) (6, N = 96) = 2.45, *P* = 0.0049). Moreover, more differences were observed when considering the time*treatment interaction. The interaction with the enrichment material significantly increased in the second period for pigs in the straw treatment compared to the first period. Whereas for pigs in the chain treatment the percentage of interaction decreased in the second and third periods compared with the first (Figure 1a, χ^2^ (4, N = 72) = 8.84, *P* = 0.009). Activity was significantly higher for pigs in the paper treatment in the first and final periods, whereas a higher activity percentage was presented in the first period compared to the other three treatments (Figure 1b, χ2^2^(6, N = 96) = 8.65, *P* = 0.009). The pigs in the wood treatment showed a decrease in activity over time (χ^2^(6, N = 96) = 8.79, *P* = 0.014). Social behaviour was higher in the first period for the pigs in the straw treatment compared with the other three treatments, whereas in the final period a higher percentage was found for pigs in the paper treatment (Figure 1c, χ^2^ (6, N = 96) = 7.10, *P* = 0.06). Interaction with the pen structures was higher for the pigs in the paper treatment compared with the other three treatments in all three periods (Figure 1d, χ^2^ (6, N = 96) = 7.82, *P* = 0.07). The eating/drinking percentage increased for the pigs in the paper and straw treatments in the third period compared with that in the first and second periods, but this tendency was not found for pigs in the chain and wood treatments (12.83 ± 6.06, 12.84 ± 6.77, 10.15 ± 5.18 and 10.50 ± 5.04, for the paper, straw, chain and wood treatments in P3, respectively, *P* = 0.06).

**Figure 2:**
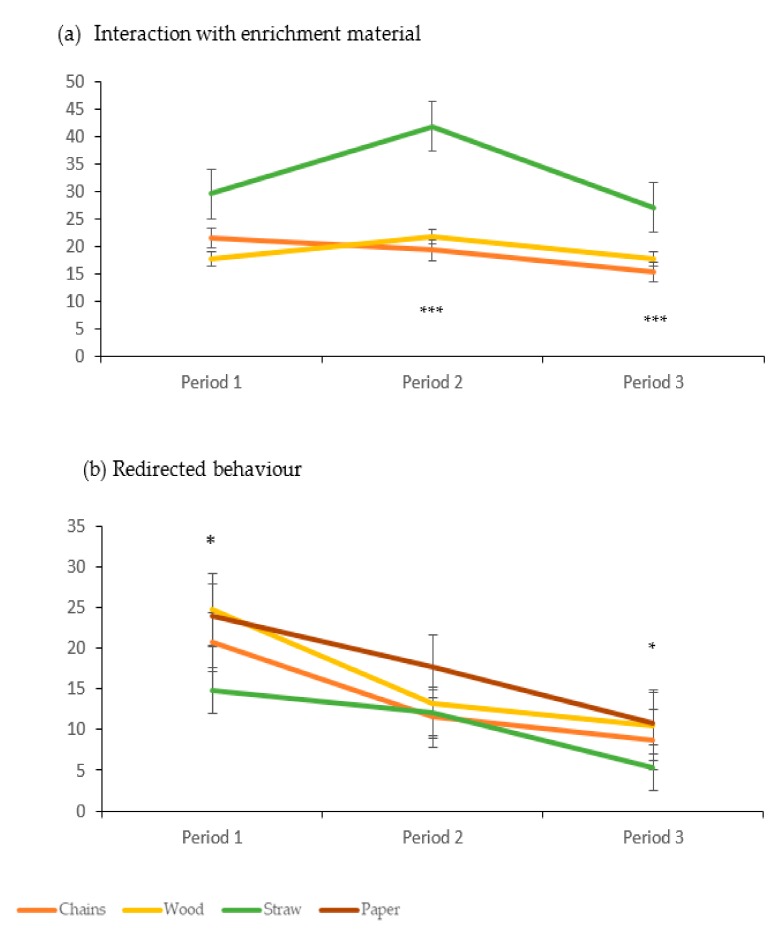
The behaviour observed by focal sampling (Mean counts and SE) for the four types of enrichment materials. 1-3: averaged observations for first, second and third month of observations. * *P* < 0.05, *** *P <* 0.001 (see text for explanation of statistical differences).

For the focal observations, the treatment*time interaction was significant for the use/interactions with the enrichment materials. This showed a decrease over the three periods of observation for pigs in the chain treatment whereas for pigs in the straw treatment, this interaction was significantly higher in the second period (F (Fisher) (4, 18) = 9.22, *P* = 0.0003, Figure 2a). For redirected behaviour, the counts were lower in the first period in the straw treatment and then they decreased, particularly between the second and third period. Whereas for the pigs in the chain and wood treatments, the decrease was only pronounced between the first and second periods (Figure 2b, F (6, 24) = 7.01, *P* = 0.025). There was an increase over time in negative social behaviour for pigs in the paper treatment (12.50 ± 5.00, 9.75 ± 2.7, 8.00 ± 4.76 and 8.50 ± 2.38, for the paper, chain, wood, and straw treatments at P3, respectively, *P* = 0.07). Sexual behaviour and positive social behaviour were not significantly affected by either enrichment treatment, time or its interaction.

Due to the low incidence, the differences in stereotypies could not be statistically analysed. However, the stereotypies were counted on 38 occasions during the focal observations, 63.5% (24 counts) in the paper treatment, 28.9% (11 counts) in the chain treatment, 5.2% in the wood treatment (2 counts) and 2.6% (one count) in the straw treatment.

### 3.2. Physiological Parameters

The CgA level was measured when the pigs were 128 days old (the first sample was taken after provision of enrichment) and was significantly higher compared to the basal level at 67 days old and the level in the last sample at 164 days old (F (2, 259) = 0.2, *P* = 0.006 Table 2). Pigs in the paper treatment showed a higher ratio of increase of CgA at 128 days old compared with that for the other three treatments (5.06 ± 4.80 vs. 2.59 ± 2.15, 1.24 ± 0.53 and 2.0 ± 2.04, for the paper, chain, wood and straw treatments, respectively, F (1, 164) = 5.31, *P* = 0.07). The N:L ratio was significantly higher in the second sample compared to the basal level before the provision of enrichment (F (1, 171) = 7.33, *P* = 0.008). There was no significant effect of fasting or interaction fasting*enrichment treatment found for the ratio N:L.

**Figure 3:**
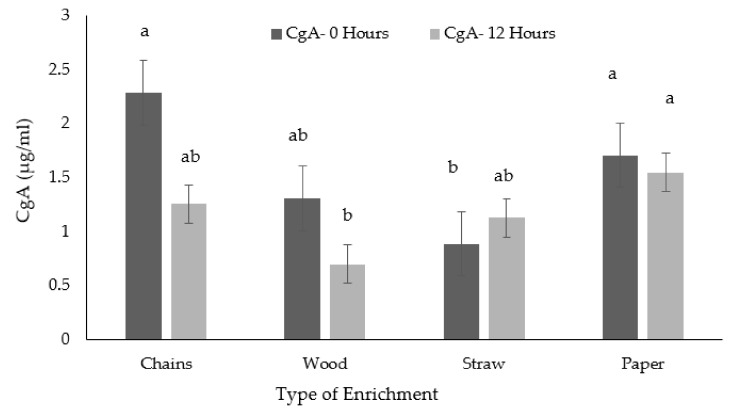
Means (and standard errors) of Chromogranin A for the 4 types of enrichment materials after the two different fasting times (0 and 12 h). Different superscripts indicate significant differences due to treatment*fasting interaction.

The pigs provided with the straw enrichment showed lower levels of CgA when subjected to 0 h of on farm fasting compared to the pigs in the other three enrichments (F (3, 85) = 1.74, *P* = 0.055, Figure 3). After 12 h of on farm fasting, the pigs in the wood treatment presented with lower levels of CgA, compared to the pigs in the paper treatment. Pigs in the chain and straw treatments presented with values between (F (3, 85) = 1.74, *P* = 0.055).

**Figure 4:**
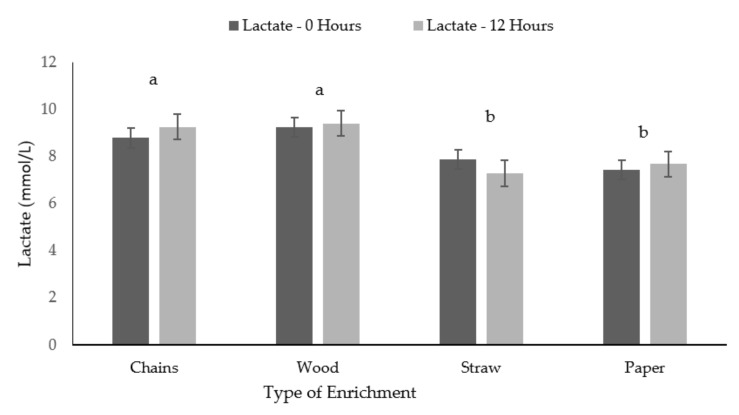
Means (and standard errors) of Lactate for the four types of enrichment materials after the two different fasting times (0 and 12 h). Different superscripts indicate significant differences due to treatment (*P* < 0.05).

The pigs provided with straw and paper presented significant lower levels of lactate compared to pigs in the chain and wood treatment, both after 0 and 12 h of on farm fasting (F (7,87) = 3.49, *P* = 0.019, Figure 4).

### 3.3. Skin Lesions

**Figure 5:**
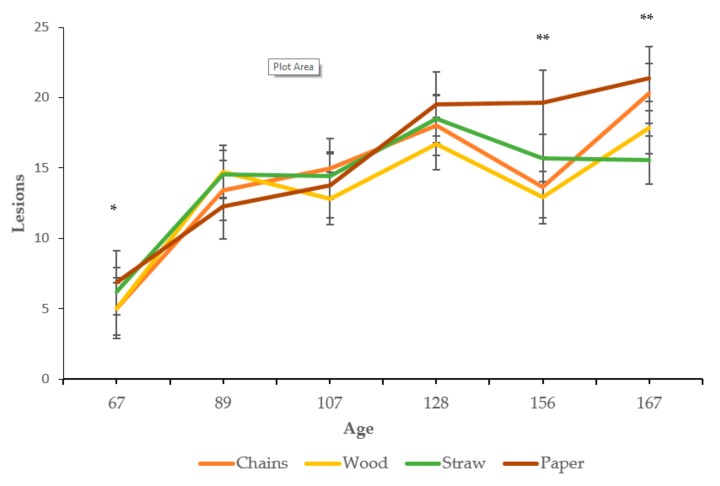
Total number of lesions (counts and standard errors) for the four types of enrichment material over time. * *P* < 0.05; ** *P* < 0.01 (see text for specific differences).

As illustrated in Figure 5, the total number of lesions significantly increased over time for all enrichment treatments (F (5, 460) = 131.78, *P* < 0.0001). The time*treatment interaction was also significant. At the first measurement (67 days old, before provision of enrichments), a significantly higher count of lesions was found for the pigs in the paper and straw enrichments, compared to those in the chain or wood treatment (F (15, 460) = 4.42, *P* = 0.049). At 156 days old, the pigs in the paper treatment presented a significantly higher count of body lesions compared to the other three treatments (F (15,460) = 4.42, *P* = 0.0027). At the last evaluation (167 days old), the pigs in the paper treatment showed a significantly higher number of lesions compared with the pigs in the chain and wood treatments which were higher than the number of lesions in the pigs who were offered straw (F (15, 460) = 4.42, *P* = 0.0099).

Concerning the number of lesions, a significant increase was observed in all the specified areas of the body over time (*P* < 0.0001), and the interaction of time*treatment was also significant for some of the areas, as follows. In the rear region (the tail was included in this study), pigs in the paper treatment presented a significantly higher count on the first basal measurement, compared to those pigs in the chain, wood and straw treatments (Table 3, F (15, 460) = 3.71, *P* = 0.021). At the final evaluation, the pigs in the chain and paper treatments showed a significantly higher count of rear lesions compared to those in the wood and straw treatments (F (15, 460) = 3.71, *P* = 0.021). At the first measurement, for the front region, the pigs in the straw treatment had a higher count of lesions compared to those in the other three treatments (F (15, 460) = 2.98, *P* = 0.07). In contrast, at the final measurement, the pigs in the straw treatment showed a significantly lower count compared to those in the other three treatments (F (15, 460) = 2.98, *P* = 0.032). For the ears, side and leg regions, the interaction time*treatment was not significant. No severe tail lesions, i.e., with fresh blood, were observed in any of the treatments at any time.

### 3.4. Weight and Productivity Data

The enrichment treatments did not significantly affect any of the productivity parameters that were evaluated (weight, average daily gain, daily feed intake and feed conversion ratio, Table 4). The weight of the pigs increased significantly over time (F (3, 36) = 282.63, *P* < 0.0001), and at the same rate for all four enrichment treatments. Overall the average daily gain (ADG), daily feed intake (DFI) or feed conversion ratio (FCR) were not significantly affected by the enrichment treatments (the average for all treatments was 0.89 ± 0.04 kg/day; 1.96 ± 0.11 kg/day and 2.21 ± 0.06, respectively). The ADG and DFI were significantly lower when the first measurement was taken (at between 68 and 90 days old), compared with the other three measurements (F(ADG) (3,36) = 18.74, F(DFI) (3,36) = 262.83, *P* < 0.0001). The feed conversion ratio was significantly lower at the first two measurements (68–90 and 91–107 days old), compared with the third and fourth measurements (F (3,36) = 103.87, *P* < 0.0001).

### 3.5. Carcass and Meat Quality Parameters

No significant effects from the enrichment treatments were found in any of the carcasses or meat quality parameters (Table 5, provides a summary of all the parameters evaluated). A significant effect of the farm fasting was found for the carcass weight, where those pigs who were fasted for 12 h had a lower carcass weight than those not fasted (F (1, 87) = 7.17, P = 0.0089).

## 4. Discussion

The aim of this study was to evaluate the effects of four different types of enrichment materials, feasible for implementation in Spanish pig housing and climate conditions, on different behavioural, welfare, productivity and meat quality indicators. The objective was to evaluate whether these different enrichment materials met the criteria of effectiveness or indicated the possibilities for success as suggested by Van der Weerd and Day and as follows [3]: (1) the enrichment material should increase species-specific behaviour, (2) it should maintain or improve levels of health, (3) it should improve the economics of the production system, and (4) it should be practical to employ. The different parameters and indicators evaluated in this study are discussed with a focus on which of the four criteria they can mostly be associated with. The authors acknowledge that they did not exhaustively investigate all the parameters that could be included in the different criteria, particularly regarding the economic impact. Therefore, criteria three and four are areas that require more research in the future.

### 4.1. Behavioural Observations: Increase or Allowance in Species-Specific Behaviour

One of the main objectives for providing enrichment materials is to enhance the possibility of pigs performing their exploratory and rooting behaviour. It has been said that pigs reared with environmental enrichment are more stimulated [3]. One of the expected behavioural outcomes of this higher stimulation is an increase in exploratory behaviour, which has been reported more in pigs in enriched environments compared to those kept in barren ones [13,27]. Moreover, pigs have been said to have a preference for chewable, destructible, rootable and deformable materials [6], and therefore, materials meeting those characteristics are considered to better satisfy the pigs’ exploratory behavioural needs. The EU Recommendation 2016 [21] classifies such materials as optimal, suboptimal or marginal based on the way they are presented and their nature (i.e., how edible, manipulable, investigable and chewable they are). The materials chosen for this study were, in accordance with the EU Recommendation 2016, marginal (chain), suboptimal (straw and paper) or suboptimal to marginal (wood attached to a chain, since classification of wood depends on its softness). The results found in this study support that, overall, straw is the material that better meets the criteria of enhancing pigs’ exploratory behaviour, which is in keeping with the findings of previous studies and expert opinions [3,17]. Both in the focal and scan observations, the pigs were found to interact more with straw in the rack, when compared to both chain (in all observations of periods two and three) and wood (in all focal observations and scans of period two). It was not possible to statistically evaluate the difference with paper, since the pigs took a mean time of 10 min to destroy, manipulate and/or chew the paper that was given to them twice a day. Therefore, almost no paper was left when the observations were carried out, but as this finding was also considered a result, it was decided to adhere to the initial design of the experiment. Paper certainly met the criteria of being manipulable, investigable and chewable, but it only enhanced the exploratory behaviour of the pigs for a short time, and the practicalities of that are discussed further in Section 4.3.2.

The findings of this study also confirm that, somehow, properly or sufficiently manipulative materials help to enhance the exploratory behaviour of pigs directed towards enrichment, and there was a significant increase in the percentage of pen interactions found for the paper treatment (i.e., the treatment with low time availability of enrichment), compared with the other three. This is in keeping with findings from previous studies, which report that pigs in barren environments channel their activities towards pen fixtures [3,27]. However, pigs in the paper treatment tended to be more active and eat more in certain periods compared to those in some of the other treatments, but with a less clear pattern than for interactions with the pen. Moreover, lying behaviour did not increase in the pigs in the paper treatment as a consequence of not being able to interact with the enrichment materials during the observations. Therefore, the scan observation results seem to suggest that when sufficient enrichment is not available, pigs may target a good part of their exploratory needs towards the pen structures. On the other hand, in the focal observations, the pigs in the paper treatment were also found to have a higher incidence of redirected behaviour towards other pigs. This result also supports previous findings that pigs reared in enriched environments show less redirected behaviour such as tail biting, ear chewing or belly-nosing, compared to those kept in barren conditions [28,29]. As previously mentioned, the exploratory behaviour of the pigs is considered out of concern for their welfare, and, consequently, pigs kept in barren conditions seem more likely to not only present with increased redirected behaviour, but also other abnormal behaviour [30].

The lowest significant percentages or counts of interaction with enrichment material were found for chains. Chains are often considered to be ‘inappropriate’ as an enrichment material, at least when they are not combined with other materials [4,21]. A recently published paper of expert opinions on whether indestructible objects are acceptable as adequate enrichment materials suggests that: ‘improving the short metal chain by making it longer, so that it reaches floor level and providing more chain ends, would significantly and considerably improve pig welfare, almost (but not quite) sufficiently to reach the experts’ cut-off point for acceptability’ [31]. The chain used in this experiment was not like that suggested by these experts, and the findings were in keeping with previous reports that showed how chains produce less exploratory behaviour compared with other more destructible materials [4,19,21]. Another important observation regarding chain is the fact that the percentage of pig interactions decreased over time to a greater extent than with straw and wood. With straw, and to a lesser extent wood, the pigs were found to interact more with the enrichment in the second period and, afterwards. However, this use decreased in the third period to the levels found in the first month, and for chain treatment the decrease was steady over time. A sense of novelty has also been reported as an important attribute for enhancing the effectiveness of enrichment materials [4,19]. This study was not designed to properly evaluate the effect of novelty itself, however the results support previous findings that the pigs’ interest in most of the enrichment materials decreased over time and was more pronounced for the chain treatment [19].

### 4.2. Physiological Parameters and Skin Lesions: Maintenance/Enhancement of Health

To discuss the effects of the four types of enrichment material on health, the indicators chosen relate not only to the immune system (neutrophil/lymphocyte ratio), but also to stress response (CgA, Lactate), and skin lesions. Moreover, the incidence of disease was also recorded as part of normal farm routines. In this sense, pigs were not affected by any pathology or disease outbreak, except for one pig treated for mild lameness. It has been suggested that when enrichment is provided as bedding, especially straw, it may have a negative impact on health, because it can harbour pathogens and increase the levels of dust in housing systems [See 3, for a review]. However, in this study the enrichment was provided as point source objects and, therefore, small differences between the different types of material were expected in relation to the risk of disease dissemination. There were no differences found in the neutrophil/lymphocyte ratio between treatments, only a significant increase over time in all treatments. Several studies have shown that increasing corticosteroids because of stress results in a redistribution of white blood cells involved in the immunological response, such as an increase in neutrophils (N) and a decrease in lymphocytes (L) [32]. For this reason, an increase in the ratio N:L has been suggested as a potential indicator of acute and even chronic stress [32]. On the other hand, previous studies report a higher N:L ratio in weaned pigs compared to fattening pigs at different ages [33]. Therefore, the increase in the N:L ratio over time found in this study could be interpreted as an indication that stress levels increased equally for all the enrichment treatments throughout the experiment and was perhaps associated with other factors such as lower space availability due to growth or higher external temperatures at the end of the experiment. This possible explanation should be interpreted with caution, because the second blood sample was obtained after exsanguination. Unlike the hormonal response to stress, the initial leukocyte response begins over a time span of hours to days depending on factors such as intensity of the stressor [32], but the effects of transport and lairage cannot be discarded, since it was not possible to obtain a baseline level before transportation took place.

CgA is an acidic soluble protein stored and co-released with catecholamines to the blood from the vesicles of the adrenal medulla, the sympathetic nerve endings ad neuroendocrine tissues [24]. Salivary CgA has been described as a good acute stress indicator in pigs [23,24], and in recently published papers there was a hypothesis that it could also be a potential indicator of chronic stress [23]. In this study, the levels of CgA found were similar to those previously reported [23,24], and no significant differences were observed between enrichment treatments in absolute values. However, the CgA levels increased in the second sample when compared with the basal levels and tended to be higher for those pigs in the paper treatment. This is partly in keeping with previous findings [23], in which a significant increase in CgA levels was observed in pigs kept in barren environments compared to those offered a combination of three enrichment materials (rope, sawdust and ball). Some authors [23] suggest that an increase in CgA over time for those pigs not offered enrichment materials could indicate chronic stress. In this experiment, the pigs in the paper treatment spent little time with the enrichment material, similar to those pigs raised with no enrichment in the previous study. However, at the end of the study the increase in the CgA ratio did not differ between treatments, indicating that perhaps the pigs in the paper treatment had adapted physiologically to their conditions. Further experiments are required to ascertain the role of a lack of sufficient and/or appropriate enrichment materials on CgA as an indicator of chronic stress response. 

The pigs in this experiment were also subjected to different pre-slaughter fasting times (0 and 12 h). The lack of on farm fasting has been described as a stressor [34] and was used to reveal whether pigs in any of the enrichment treatments had a greater capacity to cope with it. An interaction between fasting time and enrichment treatment was found for CgA, this being the pigs in the straw treatment who seemed to cope better with the more stressful situation of the lack of on farm fasting. There is limited research into the effects of providing enrichment material and the responses to different pre-slaughter conditions, and the results are inconsistent. Geverink et al. [35] found a rise in the pig’s cortisol levels due to their transportation being enriched with straw bedding. However, it was difficult to compare the results with those for pigs kept in barren environments because straw was omitted during the hours prior to transportation, and apparently, this led to high baseline cortisol levels. In this study, the enrichment materials were present until all the pigs were loaded inside the truck. This also concurs with the higher lactate levels found in the pigs in the chain treatment compared with those in the straw treatment. Increased lactate levels after stressors such as transportation are interpreted as an indicator of how the animals cope with physical stress, since muscular exercise causes a high demand for oxygen and anaerobic glycolysis [34]. It has been suggested that the signalling pathways of exercise-trained muscles repeatedly exposed to glycogen degradation and resynthesis do adapt, and, thus, a lower increase in lactate levels would be expected from more active pigs. A hypothesis for the lower levels of lactate found in the pigs in the paper and straw treatments in this experiment would be that overall, they presented with higher activity behaviours. However, further research is required, with more samples over time, to clarify the effects of different types of enrichment materials on their exercise inducing capacity and how this influences muscle adaptation to other physical stressors. 

Body lesions are included as one of the parameters assessed for the criteria of ‘good health’ in the Welfare Quality^®^ assessment protocol and are said to be a proxy indicator of aggression [25]. An interaction between time and enrichment treatment was found for the total number of lesions, and for those in the front and rear region (with tail included in this study). Whereas the baseline level for pigs in the straw treatment was higher, at the end of the study there was a greater number of skin lesions for pigs in the chain and paper treatments. This result can be partly associated with the social behaviour and activity observed, which was found to be higher in the paper treatment at the end of the study and when the pigs were provided with straw at the outset. However, this pattern was not associated with the pigs in the chain treatment. Controversial results have been reported in previous studies regarding the effects of different types of enrichment, including aggression and skin lesions. Some investigations describe reduced levels of fighting and aggression in enriched environments whereas others fail to demonstrate this [see 3, for a review]. Confounding factors such as differences in activity, breed and the baseline aggressiveness of pigs have been suggested as potential explanations for the different results. Our findings of a higher incidence of lesions in the paper treatment supports the assumption that deficient or insufficient enrichment materials can enhance competition and, in turn, increase aggression. This is supported by the higher levels of negative social behaviour seen in the focal observations. Moreover, it can be speculated that the higher number of lesions on the rear region of the pigs in the paper treatment is associated with the higher percentage of redirected behaviour, although a more precise scoring system for tail damage is necessary to ascertain this. Our findings are also in agreement with previous studies reporting a reduction in aggression and skin lesions when pigs are offered straw [3,14,17]. However, it is also important to comment on other factors that are common in experimental conditions, such as the lower number of pigs per pen, the positive human-animal relationship, the reduced mixing practices and the high environmental standards when compared to commercial conditions. All of these factors could have influenced the absence of severe lesions and the lack of greater differences between the enrichment treatments in this study.

### 4.3. Areas for Future Research

#### 4.3.1. Productivity Indicators and Carcass and Meat Quality: The Economics of the Production System

In this study, no differences were found in either the performance parameters or the quality of the carcass and meat between the four types of enrichment materials. Some objects like chains, balls, metal bars, nutritious objects or cloth strips have been reported to have no influence on productivity in grower finishing pigs, which is in keeping with our results [3,36]. On the contrary, some other investigations have found better growth gains, feed intakes or feed conversion ratios, when providing point source enrichment-objects [3,13,15]. However, in most of these studies, it is important to note that the control pigs were kept in barren environments and the other pigs were provided with at least one type of enrichment, whereas in this study all the pigs were offered enrichment. Moreover, some studies have attributed the better performances found for straw bedded systems to the higher levels of activity and exploratory behaviour of the pigs which leads to higher feed intakes. The increase in the levels of exploration by the pigs with straw as bedding can be up to twenty times higher than those with objects [3]. If the underlying cause of better performance is its association with increased activity and exploration, the results obtained in this study could be explained by the increase in activity for pigs in the paper and straw treatment not being sufficient enough to trigger an increase in feed intake.

On the other hand, there were no major differences found in this study between the different enrichment materials when it came to carcass and meat quality, which concurs with previous investigations [35,37]. However, other studies have reported some effects of enriched environments such as a better water-binding capacity and lower shear force, but with no differences in pH values as found in this study [27]. Previous studies also reveal that the ease of handling and behaviour in a novel environment can be influenced by the environmental enrichment [16,35]. As previously discussed, the pigs provided with straw showed significantly lower levels of CgA at slaughter than those offered chain, but none of the carcass or meat quality parameters were affected. It remains to be discovered whether the fasting treatments were sufficient or insufficient as pre-slaughter stressors and affect meat quality, or whether the source objects used can be expected to improve meat quality.

Regarding the cost of the treatments, straw in a rack requires the highest investment and maintenance cost. It is estimated that a mean quantity of 30 g per day per pig of straw was used. This is higher than the quantity of 8.3 g per day per pig reported in a recent study for chopped straw [38]. The same author reports higher amounts in previous trials, which agrees with the results of this study [19]. The differences may be due to the length of straw provided (non-chopped was used in this study) or to the design of the rack system. Therefore, a reduction in the amount of straw and, thus, in the costs could be achieved by considering these aspects. A proper economic evaluation, considering the inputs and outputs is an important area for future research.

#### 4.3.2. Practicalities to Employ

The chain enrichment was chosen for the control group in this study (chain is not appropriate on its own) or because if proven to work, a combination could be easily implementable on Spanish pig farms. Of the four materials tested, paper seemed to trigger pigs’ interest, but only for a short time and providing paper more often would be time consuming for farmers. Another concern in relation to newspaper regards the potential toxicity of the ink. As paper is commonly used in some farms, it was used in this study to obtain more information and there appeared to be no detrimental effect on productivity. Chopped and pelleted newspaper has previously been considered safe as animal bedding [39]. Moreover, previous studies did not find any detrimental effect on pre-weaning mortality and weaning weight when using newspaper as enrichment material for piglets [40]. However, it must be pointed out that there is still insufficient information on whether ingesting newspaper is a risk to pigs. This depends on the type of ink used as some do emit volatile compounds such as toluene. Therefore, even if the practical issues previously mentioned can be overcome, more information is required before recommending the use of paper including that which uses new soy and water-based inks. The wood provided was renewed, and softer wood is recommended due to its manipulability and interest to pigs. Finally, the straw in a rack was found to be the most interesting for pigs and practical. The people topping it up regularly said it increased labour time to approximately 15 min a day. However, a major constraint of using straw was highlighted when the slatted floor was lifted at the end of the experiment. A considerable amount of straw was found mixed with the slurry, creating a risk of blockage in the system. Therefore, it is strongly recommended to improve the design of the rack by adding a tray underneath to prevent this problem, or to consider other straw lengths.

## 5. Conclusions

Straw in a rack is the material found to enhance more species-specific behaviour, such as exploratory behaviour towards the enrichment. In addition, the pigs provided with straw presented with the lowest percentage of redirected behaviour. Chains presented a more pronounced reduction in pigs’ interest over time when compared with straw. Paper was only available for a short period after it was provided, and the percentage of redirected behaviour, either towards the pen or to other pen-mates as well the values of CgA in saliva tended to be higher. No major health problems could be attributed to any of the enrichment treatments. Pigs offered straw presented with a lower increase in CgA levels when subjected to a more stressful pre-slaughter treatment (no fasting) when compared with the pigs provided with chains and paper, potentially indicating a greater capacity to cope with this stressful condition. Lactate levels were found to be lower in the pigs in the straw and paper treatments, the ones in which pigs presented with higher levels of on farm activity, which may have allowed them to cope better with physical stress. Skin lesions were lower for the pigs in the straw treatment compared to pigs offered paper, who also had a higher percentage of negative social behaviour. The cost of providing any of the enrichments in this study was not that expensive, although the straw in a rack had a higher labour cost and management expenses. In this study, the economic benefits of providing any of the enrichment materials tested would have to be linked to the potential advantages for animal welfare, because no positive effects on performance or meat quality were found. To summarise, straw in a rack proved to be the best option from an animal welfare point of view. However, some design aspects need to be improved, such as adding a tray to collect the spare straw, to avoid the risk of blocking the slurry systems. It is important to note that the overall welfare status of the pigs in these experimental conditions was good which may have balanced out any differences between the enrichments provided.

## Figures and Tables

**Table 1:** Ethogram of the Behaviours Evaluated in the Scans or Focal Samplings taken over 12 weeks of Observations.

Specific Category	Definition
*SCAN SAMPLING* *Activity*	
Standing inactive	Pig is upright on all four legs, neither moving forward or backward
Walking	Pig is upright on all four legs, and moves in the pen
Sitting inactive	Pig is upright on two front legs, and hindquarter
Lying	Pig is recumbent on its belly or side
*Behaviour*	
Eat/Drink	Head or snout over bowl or trough
Interaction with the pen	Licking, chewing, nosing or sniffing unanimated objects from the pen, excluding enriched material
Interaction with enrichment material	Interaction with each type of enrichment material
Social interaction	Head or snout in contact with another pig (includes any positive or negative social behaviour)
Other behaviour	Behaviour different to that previously described
*FOCAL SAMPLING*	
Positive social behaviour	Head or snout in contact with another pig. The receiver doesn’t react negatively
Negative social behaviour	Aggression, fights to another pig with a negative response from the receiver
Stereotypies	Stereotypical behaviour, basically animal rubs the bars of the crate with the mouth, and other stereotypical behaviour
Redirected behaviour	Vertical rub movements with the snout to the belly of a pen-mate (belly nosing), bite or suck the ear of a pen-mate (ear biting) and bite or suck the tail of a pen-mate (tail biting)
Interaction with enrichment material	Interaction with each type of enrichment material
Sexual behaviour Other behaviour	Sexual behaviour, one animal mounting or trying to mount another pig Any other behaviour

**Table 2:** The Mean (and standard deviations) of the various physiological indicators of welfare for the 4 types of enrichment over time.

	Enrichment Material Treatment					
	*Chain*	*Wood*	*Straw*	*Paper*	P Treatment ^3^	P Time
CgA 67 ^1^	0.77 ^a^ ± 0.87	0.73 ^a^ ± 0.99	1.10 ^ab^ ± 1.57	1.08 ^a^ ± 1.57		
CgA 128	1.15 ^b^ ± 2.20	1.09 ^ab^ ± 1.64	1.34 ^b^ ± 2.19	2.51 ^b^ ± 3.89	NS	**
CgA 164	0.73 ^a^ ± 0.61	0.72 ^a^ ± 0.52	0.63 ^a^ ± 0.57	0.71 ^a^ ± 0.44		
N:L 66 ^2^	0.66 ^a^ ± 0.32	0.67 ^a^ ± 0.24	0.67 ^a^ ± 0.32	0.62 ^a^ ± 0.21	NS	**
N:L 170	0.79 ^b^ ± 0.31	0.83 ^b^ ± 0.31	0.82 ^b^ ± 0.45	0.77 ^b^ ± 0.47		

^1^ CgA 67, 128, 164 = Chromogranin A (µg/ml) at 67, 128 or 164 days old. ^2^ N:L 66, 170 = Neutrophil/Lymphocyte ratio at 66 or 170 days old. ^3 a^, ^b^ and ^c^: means with different superscripts present significant differences due to treatment (row comparisons) or due to time (column comparisons). ** = *P* < 0.01.

**Table 3:** Counts of lesions (mean and standard error) in the rear and front regions before the provision of the enrichment treatments (67 days old) and at the final evaluation (167 days old).

	Enrichment Material Treatment				
	*Chain*	*Wood*	*Straw*	*Paper*	P Treatment * Time ^1^
Rear Lesions 67	0.62 ^a^± 0.41	1.08 ^a^ ± 0.89	0.63 ^a^ ± 1.57	1.83 ^b^ ± 1.22	
Rear Lesions 167	7.20 ^d^ ± 2.90	6.00 ^c^ ± 1.85	5.66 ^c^ ± 2.66	7.17 ^d^ ± 2.62	*
Front Lesions 67	1.87 ^a^ ± 0.70	1.71 ^a^ ± 0.56	2.29 ^b^ ± 1.83	1.79 ^a^ ± 0.56	*
Front Lesions 167	6.2 ^d^ ± 4.9	5.25 ^d^ ± 3.35	3.95 ^c^ ± 3.01	5.62 ^d^ ± 4.48	

^1^ Different superscripts indicate significant differences due to treatment*time interaction.

**Table 4:** The Mean (and standard deviations) of Average Daily Gain, Daily Feed Intake and Feed Conversion Ratio for the four types of enrichment material at different periods (1 to 4) and of the initial Weight and Final Weight.

	Enrichment Material Treatment					
	*Chain*	*Wood*	*Straw*	*Paper*	P treatment ^3^	P time
ADG 1 ^1,2^	0.77 ^a^ ± 0.07	0.77 ^a^ ± 0.03	0.75 ^a^ ± 0.04	0.74 ^a^ ± 0.06	NS	***
ADG 2	0.92 ^b^ ± 0.08	0.91 ^b^±0.13	0.91 ^b^ ± 0.15	0.92 ^b^ ± 0.068		
ADG 3	0.93 ^b^ ± 0.11	0.92 ^b^±0.04	0.91 ^b^ ± 0.02	0.92 ^b^ ± 0.05		
ADG 4	0.94 ^b^ ± 0.07	0.97 ^b^ ± 0.05	0.98 ^b^ ± 0.04	0.95 ^b^ ± 0.09		
DFI 1	1.39 ^a^ ± 0.08	1.41 ^a^ ± 0.06	1.44 ^a^ ± 0.07	1.43 ^a^ ± 0.02	NS	***
DFI 2	1.72 ^b^ ± 0.14	1.63 ^b^ ± 0.037	1.71 ^b^ ± 0.19	1.76 ^b^ ± 0.15		
DFI 3	2.14 ^b^ ± 0.19	2.21 ^b^ ± 0.16	2.18 ^b^ ± 0.16	2.15 ^b^ ± 0.11		
DFC 4	2.45 ^b^ ± 0.19	2.51 ^b^ ± 0.15	2.56 ^b^ ± 0.15	2.48 ^b^ ± 0.13		
FCR 1	1.80 ^a^ ± 0.07	1.83 ^a^ ± 0.03	1.90 ^a^ ± 0.16	1.93 ^a^ ± 0.13	NS	***
FCR 2	1.87 ^a^ ± 0.12	1.86 ^a^ ± 0.22	1.90 ^a^ ± 0.11	1.94 ^a^ ± 0.12		
FCR 3	2.32 ^b^ ± 0.15	2.41 ^b^ ± 0.14	2.40 ^b^ ± 0.19	2.34 ^b^ ± 0.07		
FCR 4	2.62 ^b^ ± 0.22	2.59 ^b^ ± 0.24	2.61 ^b^ ± 0.10	2.61 ^b^ ± 0.24		
IW	25.60 ^b^ ± 1.61	27.14 ^b^ ± 1.71	27.04 ^b^ ± 1.54	26.20 ^b^ ± 0.48		
FW	105.94 ^b^ ± 6.26	107.96 ^b^ ± 4.12	106.43 ^b^ ± 4.63	106.16 ^b^± 4.02	NS	***

^1^ ADG: Average Daily Gain (in kg/day); DFC: Daily Feed Intake (in kg/day); FCR: Feed Conversion Ratio; IW = weight at 68 days of age (in kg); FW = weight at 157 days of age in kg. ^2^ Periods (in days of age) = 1: 68-90; 2: 91-107; 3: 108-129; 4: 130-157. ^3^ Means with different superscripts present significant differences due to treatment (row comparisons) or due to time (column comparisons). ***: *P* < 0.001; NS = not significant. Interaction treatment * time = NS.

**Table 5:** Means (and standard deviations) of carcass and meat quality parameters for the four types of enrichment material after implementing two different fasting times (0 and 12 h).

	Enrichment Material Treatments				
	*Chain*	*Wood*	*Straw*	*Paper*	P Fasting ^2^
Carcass weight 0 h (kg) ^1^	88.98 ^a^ ± 2.83	87.61 ^a^ ± 2.72	88.46 ^a^ ± 2.71	87.83 ^a^ ± 2.71	*
Carcass Weight 12 h (Kg)	81.36 ^b^ ± 2.71	85.13 ^b^ ± 2.71	82.73 ^b^ ± 2.71	82.99 ^b^ ± 2.72	
Carcass yield (%) ^2^	74.50 ± 0.51	74.71 ± 0.44	74.49 ± 0.48	74.09 ± 0.49	NS
Carcass Lean percentage (%)	59.05 ± 0.68	58.79 ± 0.65	58.46 ± 0.64	59.27 ± 0.65	NS
pHu SM	5.48 ± 0.02	5.50 ± 0.09	5.50 ± 0.019	5.52 ± 0.019	NS
ECu SM	7.56 ± 0.3	8.03 ± 0.28	8.07 ± 0.27	7.88 ± 0.28	NS
L* SM	49.57 ± 0.61	50.27 ± 0.58	49.79 ± 0.57	49.59 ± 0.58	NS
a* SM	6.15 ± 0.36	5.74 ± 0.34	6.13 ± 0.34	6.31 ± 0.34	NS
b* SM	0.12 ± 0.1	−0.19 ± 0.1	0.08 ± 0.1	−0.07 ± 0.1	NS

^1^ Fasting times = 0 or 12 h. ^2^ Carcass lean percentage obtained using the Fat-O-Meat’er; pHu SM: muscle pH at *Semimembranosus (SM)* 24 h p.m.; ECu SM=electrical conductivity measured using the *Semimembranosus*; L*, a*, b*: Luminosity, redness and yellowness.^3^ * *P* < 0.05; the Means for carcass weight with different superscripts are significantly different due to fasting (column comparisons). No other significant effects were observed either for enrichment material treatment (row comparisons) or for interaction Enrichment*Fasting.
